# Evaluation of an Aboriginal and Torres Strait Islander strengths based coaching program: a study protocol

**DOI:** 10.1186/s12889-021-11503-3

**Published:** 2021-07-23

**Authors:** Alison Brown, Fiona Mensah, Graham Gee, Yin Paradies, Samantha French, Lea Waters, Kerry Arabena, Gregory Armstrong, Jan Nicholson, Stephanie J. Brown, Kelsey Hegarty, Rebecca Ritte, Kristy Meiselbach, Margaret Kelaher

**Affiliations:** 1 University of Melbourne, Melbourne, Australia; 2 Murdoch Children’s Research Institute and University of Melbourne, Melbourne, Australia; 3 Murdoch Children’s Research Institute, Melbourne, Australia; 4 Deakin University, Burwood, Australia; 5 Aboriginal Housing Victoria, Fitzroy North, Australia; 6 Karabena Consulting, Melbourne, Australia; 7 La Trobe University, Bundoora, Australia

**Keywords:** Protocol, Evaluation, Aboriginal and Torres Strait Islander, Strengths-based, Coaching, Prospective cohort

## Abstract

**Background:**

Increasingly, strength-based approaches to health and wellbeing interventions with Aboriginal and Torres Strait Islander Australians are being explored. This is a welcome counter to deficit-based initiatives which can represent a non-Indigenous view of outcomes of interest. However, the evidence base is not well developed. This paper presents the protocol for evaluating a strengths-based initiative which provides life coaching services to Aboriginal and Torres Strait Islander community housing tenants. The study aims to evaluate the effect of life coaching on social and emotional wellbeing (SEWB) of tenants in three Victorian regions.

**Methods:**

The More Than a Landlord (MTAL) study is a prospective cohort study of Aboriginal Housing Victoria tenants aged 16 years and over that embeds the evaluation of a life coaching program. All tenant holders in one metropolitan and two regional areas of Victoria are invited to participate in a survey of SEWB, containing items consistent with key categories of SEWB as understood and defined by Aboriginal and Torres Strait Islander peoples, and key demographics, administered by Aboriginal and Torres Strait Islander peer researchers at baseline, 6 and 18 months. Survey participants are then invited to participate in strengths based life coaching, using the GROW model, for a duration of up to 18 months. Indigenous life coaches provide tenants with structured support in identifying and making progress towards their goals and aspirations, rather than needs. The study aims to recruit a minimum of 200 survey participants of which it is anticipated that approximately 73% will agree to life coaching.

**Discussion:**

The MTAL study is a response to Aboriginal and Torres Strait Islander community and organisational requests to build the evidence base for an initiative originally developed and piloted within an Aboriginal controlled organisation. The study design aligns with key principles for research in Indigenous communities in promoting control, decision making and capacity building. The MTAL study will provide essential evidence to evaluate the effectiveness of strengths-based initiatives in promoting SEWB in these communities and provide new evidence about the relationship between strengths, resilience, self-determination and wellbeing outcomes.

**Trial registration:**

This trial was retrospectively registered with the ISRCTN Register on the 12/7/21 with the study ID:ISRCTN33665735.

## Background

Aboriginal and Torres Strait Islander peoples have inhabited Australia, for over 65,000 years [1]. Aboriginal and Torres Strait Islander peoples have, over time, developed complex societies, knowledges and cultures. Sophisticated local knowledge systems in pharmaceuticals, ecology, geography, farming and social systems along with spiritual and artistic endeavours are just a few of the skills that have enabled Aboriginal and Torres Strait Islander peoples to adapt and thrive in changing environmental conditions prior to colonisation [2, 3].

Aboriginal and Torres Strait Islander knowledges inform a broad and holistic understanding of health and wellbeing that encompasses mental, physical, cultural and spiritual health [4]. The term social and emotional wellbeing (SEWB) is used in this study to describe the health and wellbeing of Aboriginal and Torres Strait Islander peoples. In this context, SEWB describes a ‘physically healthy, culturally intact and spiritually connected person’ ([5] p.9). The term captures the way in which Aboriginal and Torres Strait Islander people view the need for a multiplicity of elements to be in balance at both a community and individual level to achieve health and wellbeing [4–7]. Gee et al. [8] have identified seven elements of Aboriginal and Torres Strait Islander social and emotional wellbeing (SEWB); these include connection to body; mind and emotions; family and kinship; community; culture; country; and spirit, spirituality and ancestors. These elements of wellbeing for Aboriginal and Torres Strait Islander people, families and communities are influenced by broader historical, political and social determinants of health. For example, against the backdrop of colonisation the extent to which communities have managed to maintain cultural continuity, self-determination and community control, will in turn shape environments that people are born into with regards to family/community stability, cohesion and wellbeing - or conversely, historical/cultural loss and social disadvantage.

For many, colonisation has seen the destruction or fragmentation of family and community, traditional lands, languages and cultural practices [9]. These are key elements of SEWB and such losses have, in many cases, resulted in profound trauma for Aboriginal and Torres Strait Islander peoples [10]. The strength, resilience and political will of Aboriginal and Torres Strait Islander peoples have enabled their survival. However, trauma associated with colonisation, assimilation practices, forced removal, ongoing structural inequities and racism persist [11]. The impact of trauma is reflected in high rates of unemployment, incarceration, substandard, insecure and overcrowded housing, rates of chronic disease, mental health conditions, suicide and hospitalisation [12–15]. Yet, these statistics only paint part of the picture.

Much research, government discourse and supporting data has had a focus on ‘difference, disparity, disadvantage, dysfunction and deprivation’ of Aboriginal and Torres Strait Islander people compared to non-Indigenous populations ( [16] p.235). As Walter and Suina [16] argue, this representation is informed by the world view of the researcher, historically a non-Indigenous worldview. While, there is a need to acknowledge the extent of disadvantage experienced by Aboriginal and Torres Strait Islander people, when this becomes the dominant narrative, it excludes alternative approaches to thinking about Aboriginal and Torres Strait Islander health and wellbeing [17]. Deficit-based approaches have consistently failed to address the current challenges experienced by Aboriginal and Torres Strait Islander peoples. In particular, they often do not adequately address and strengthen key elements of SEWB, as previously defined.

Strengths based approaches to research with Aboriginal and Torres Strait Islander people have increasingly attracted attention (see for example [18–20]). Such approaches are drawn from multiple sources, including the field of positive psychology [17]. Strengths are positive capabilities, found in individuals, which support development, growth and the attainment of goals [21]. A strengths based approach is based on the premise that inherent strengths and resilience can by unlocked with structured support. This approach recognises that individuals live within families, communities and a culture that also have strengths and resources that will contribute to wellbeing [22]. The process involves, in collaboration with a practitioner, developing self-determined goals, identifying opportunities, capabilities and assets that foster progress and growth, examining what is going well and building on it. The aim is to raise awareness of and cultivate identified strengths and assist individuals in transferring learnings to other areas of their lives and create growth for the family unit as a whole [23, 24].

Extensive national consultations with Aboriginal and Torres Strait Islander communities have identified the need to recognise collective strengths such as connection to culture and kinship systems, and resistance and commitment to unresolved social justice issues (e.g., dispossession of land, racism). Hence, strength-based approaches align with principles of Aboriginal and Torres Strait Islander empowerment, healing and self-determination [25]. They are also seen as a counter to deficit-based discourse [26].

Concurrently, critiques of strengths-based approaches warn against shifting responsibility for structural inequities to the local and individual level [27]. Further critiques highlight the western ethnocentric individualistic nature of strengths based approaches that attempt to universalise construction of self and behaviour [10, 28]. In light of these critiques, Aboriginal and Torres Strait Islander perspectives are central to adopting strength-based approaches within these communities. Specific strengths and outcomes valued by Aboriginal and Torres Strait Islander people need to be addressed.

This paper outlines the design and methods to be used in an evaluation study of a strengths-based approach to improving the SEWB of tenants of Aboriginal Housing Victoria (AHV), embedded within a prospective cohort study. This study builds on an earlier AHV pilot of a life coaching service informed by a household survey developed with families who had AHV tenancies. The pilot study addressed the SEWB of tenants by supporting their aspirations rather than needs. Aspirations were fed back to AHV and enabled life-coaches to engage with tenants to make aspiration plans and to connect tenants with appropriate services and local programs aligned to their goals. The strengths-based pilot focused on empowerment and self-determination. The pilot study provided important data to inform and influence AHV housing policy, decisions and service response. The current study, known as More Than a Landlord (MTAL), has been developed in response to calls from Aboriginal and Torres Strait Islander people and providers locally and more broadly for a rigorous evidence base to be developed through evaluation to underpin the life coaching service.

## Methods/design

This research aims to evaluate the effect of a regionally tailored, Indigenous-led life-coaching service on the SEWB of Indigenous people in three regions of Victoria. The evaluation study will be conducted in two parts examining the implementation of life coaching using qualitative and quantitative methods. Part one is an observational study of SEWB, physical health, housing, education, and financial wellbeing, using self-reported measures in a survey completed by Aboriginal and Torres Strait Islander people who are tenants of AHV who adopt life coaching and those who do not. Part two is a complimentary qualitative and quantitative study that seeks to understand the experience and impact of life coaching on tenants through a periodic pulse check and an interview. The study will contribute to building the evidence base for strengths-based initiatives in improving the SEWB of Aboriginal and Torres Strait Islander people.

MTAL is overseen by a governance group of chief investigators and AHV representatives who meet periodically. A memorandum of understanding was developed between AHV and the University of Melbourne and reviewed by the AHV board before sign off.

### Study population

**Fig. 1:**
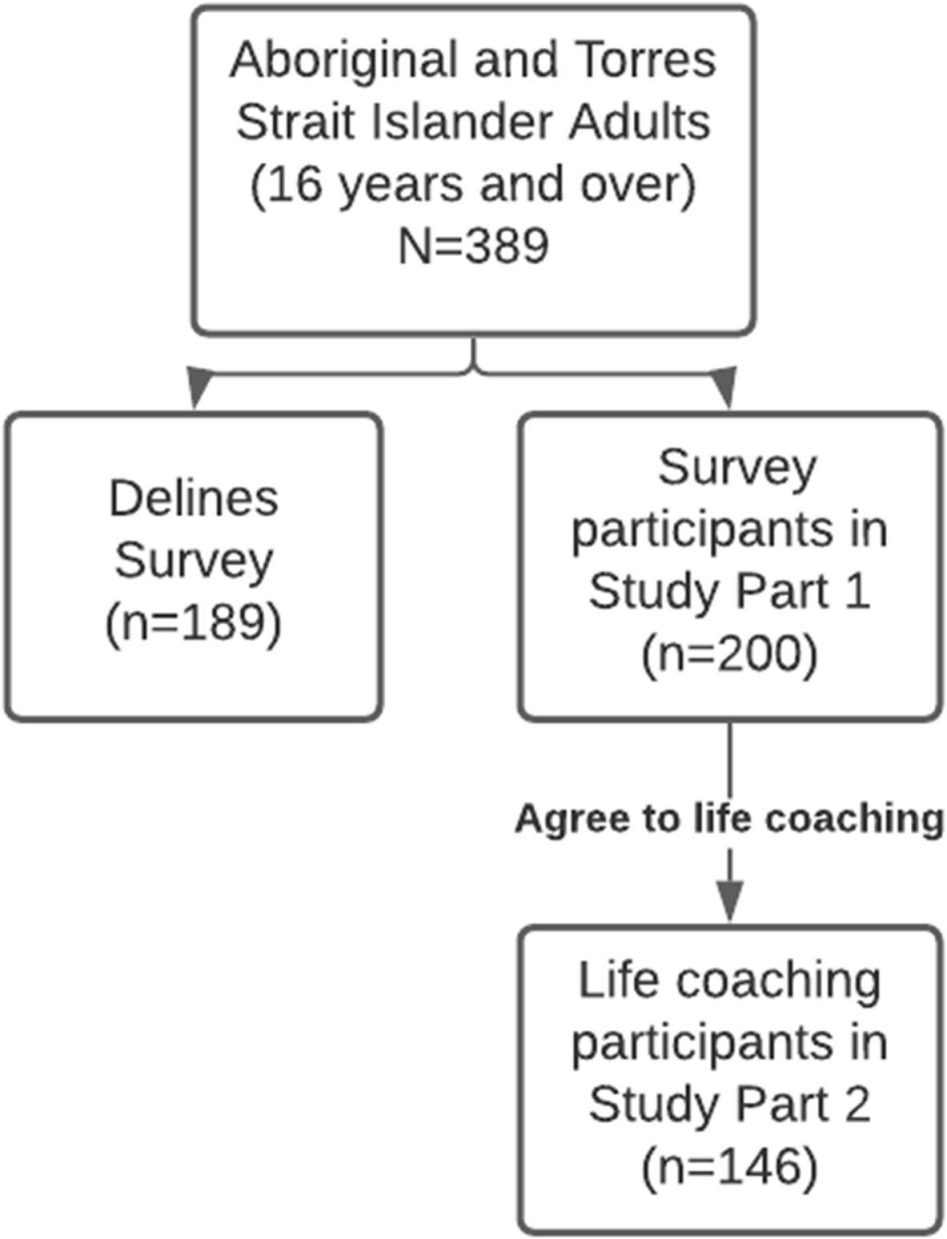
Participants type and anticipated numbers

This study is conducted in partnership with AHV, which is an Aboriginal community organisation responsible for managing over 1500 rental properties for Aboriginal and Torres Strait Islander people living in Victoria, Australia. AHV has identified three regions in which the study will be conducted: North west metropolitan Melbourne and two regional areas; Ballarat and the greater Geelong area. The regions were selected by AHV based on data that indicated that the population of tenants in the regions experienced more stability in managing tenancies (e.g. fewer issues with rental payments) and may be more able to participate in life coaching. All Aboriginal and Torres Strait Islander people aged 16 years and over, who are tenants of AHV and reside in the three regions are eligible to participate. There will be three main groups of people in the research, those who decline to be surveyed, survey participants (Part 1 sample), and a subset of survey participants who become life coaching participants (Part 2 sample) as shown in Fig. 1.

Based on the uptake in the pilot survey, of the 389 tenant holders we approach in the three regions, we are anticipating that a minimum of 51% will agree to the survey. We are expecting 146 of the 200 tenant holders surveyed (73%) to continue on to life coaching. There may be additional tenants in each household recruited to the life coaching and/or survey.

### Recruitment

This section outlines the approach to recruiting study participants, and recruiting and training peer researchers and life coaches.

#### Survey and life coaching participants

Prior to recruitment, the study will be publicised through existing AHV channels such as newsletters and community events and modified to meet the requirements of COVID-19 restrictions. All named tenant holders of AHV tenancies in the three participating regions will be contacted by phone by Aboriginal and Torres Strait Islander peer researchers and invited to participate in three surveys of SEWB at baseline, 6 months and 18 months. An appointment will be made to provide and explain the survey plain language statement, obtain written informed consent and undertake the baseline survey with those agreeing to participate. This appointment may be face to face, by phone or virtually via video link.

After completing the survey, participants will be offered life coaching. Survey participants interested in undertaking life coaching will be contacted by an Aboriginal or Torres Strait Islander life coach to explain the life coaching program and make an appointment. This appointment will be face to face, unless COVID-19 restrictions require the use of phone or videolink. The life coach will provide and explain the life coaching plain language statement, obtain written informed consent and commence life coaching with those agreeing to participate.

#### Peer researchers

Approximately 10 Aboriginal or Torres Strait Islander peer researchers will be recruited by AHV from the AHV tenant population in the three regions. Drawing peer researchers from local communities is an important element of the study in both facilitating better participant engagement in the surveys and strengthening community capacity.

Positions will be advertised in AHV newsletters and through community events. An information pack will be made available to all potential applicants and will include information on the project, work expectations and the application process. Successful applicants will then be provided with a 1 week training program in research, ethics and consent, confidentiality, communicating the research to participants, contacting participants, conducting the survey, use of survey technology, participant distress protocols and preparation for work. Throughout the survey periods, peer researchers will meet weekly with a peer researcher coordinator to schedule appointments, discuss issues, problem solve and undertake any additional training.

#### Life coaches

Aboriginal and Torres Strait Islander life coaches will comprise existing AHV life coaching staff and newly recruited staff. Recruitment will target individuals with a relevant qualification in community development, health/wellbeing or life coaching. All staff will undertake a one-week course providing them with essential tools for life coaching. Training will cover coaching models, dealing with challenging conversations, developing goals and actions, cultural determinants of SEWB, introduction to research and data collection, relationship building and self-care. The training is similar in content to accredited life coaching courses, at an introductory level, and tailored to be culturally appropriate and address research requirements. The life coaches will also attend the peer researcher training.

### Study components

**Fig. 2:**
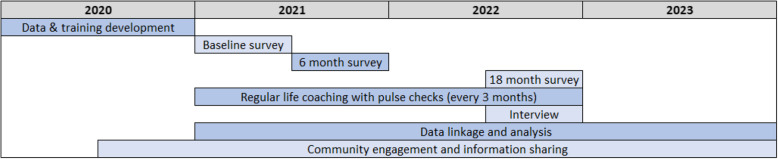
Timeline of study activites

The MTAL study consists of the following main components: development, the life coaching intervention and data collection. These study activities are described in this section and presented in the timeline in Fig. 2.

#### Development

The initial development phase consists of developing data collection tools and processes and training for peer researchers and life coaches. A survey was developed to examine SEWB in part one of the study. Data collection tools, developed for part two of the study, capture the experience and impact of life coaching and information about strengths identified, include the support plan, pulse check and a qualitative interview.

#### Life coaching intervention

The approach to life coaching will be based on the GROW (Goal, Reality, Options, Will) model [29] and motivational interviewing [30]. Life coaches will meet with life coaching participants in weekly face to face sessions for the first 4 weeks. After that, contact will be approximately fortnightly for a duration of up to 18 months via a range of formats including face to face, text, email, phone call and video link.

**Fig. 3:**
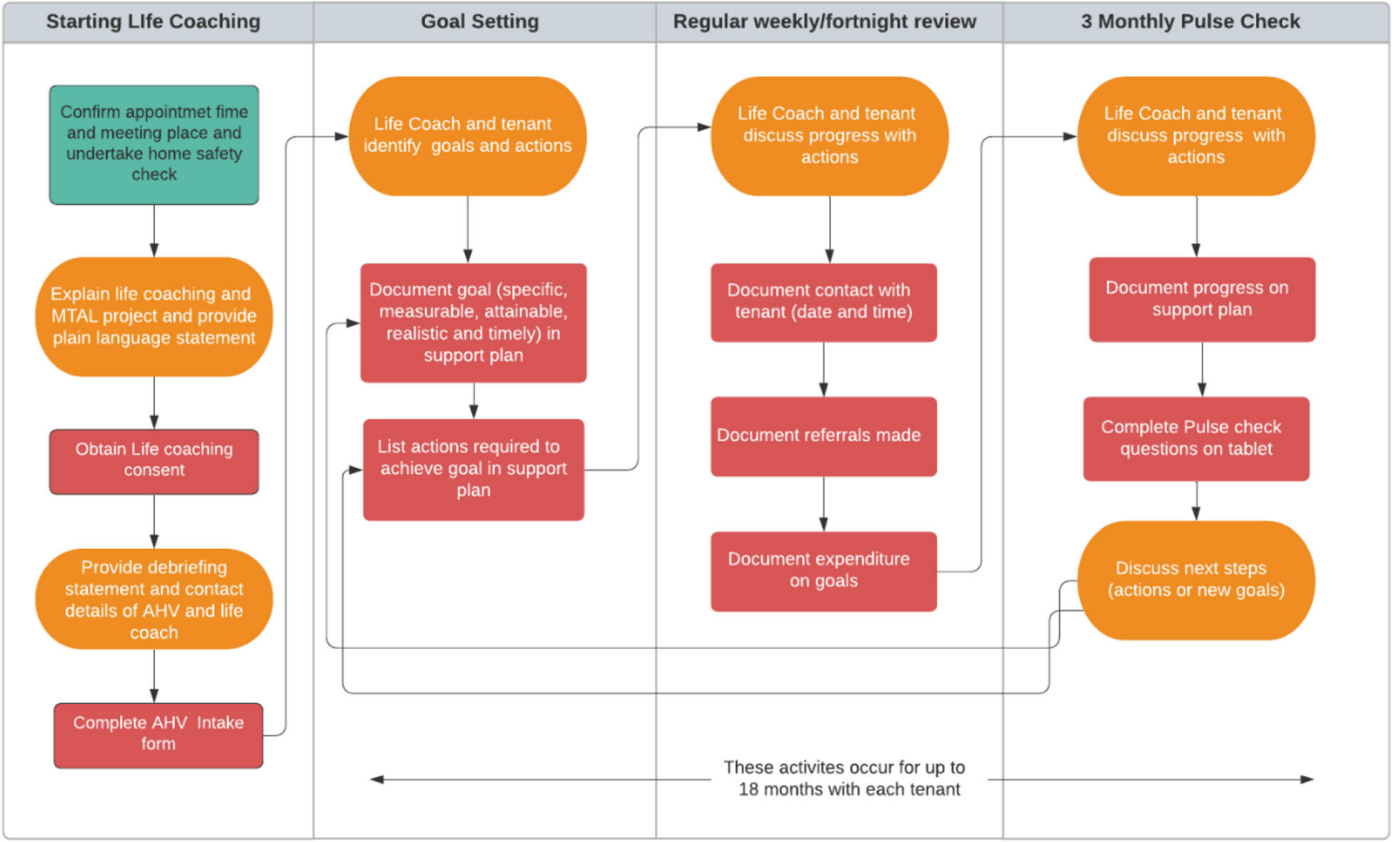
Life coaching flow chart

In the early stages life coaches will assist participants to identify and prioritise goals and identify actions required to meet goals. Life coaches will use questions derived from the GROW model to assist participants in identifying a goal they wish to work on, resources and barriers, possible actions to achieving their goal and their motivation to change [29]. Goals and actions will be documented on an electronic support plan at least every 3 months. Progress with reaching goal/s will be discussed at every session and every 3 months the life coach will update the support plan and administer a pulse check, which will prompt the tenant to reflect on their progress and their strengths. The aim of life coaching is to raise a tenant’s awareness of the strengths they possess and the resources available in the community to achieve desired changes in their life. People who have knowledge of and use their strengths has been shown to experience greater wellbeing [21]. Life coaches will meet regularly with the life coach coordinator to discuss individual coaching interventions and coaching approaches. The life coaching intervention is summarised in Fig. 3.

#### Data collection

**Fig. 4:**
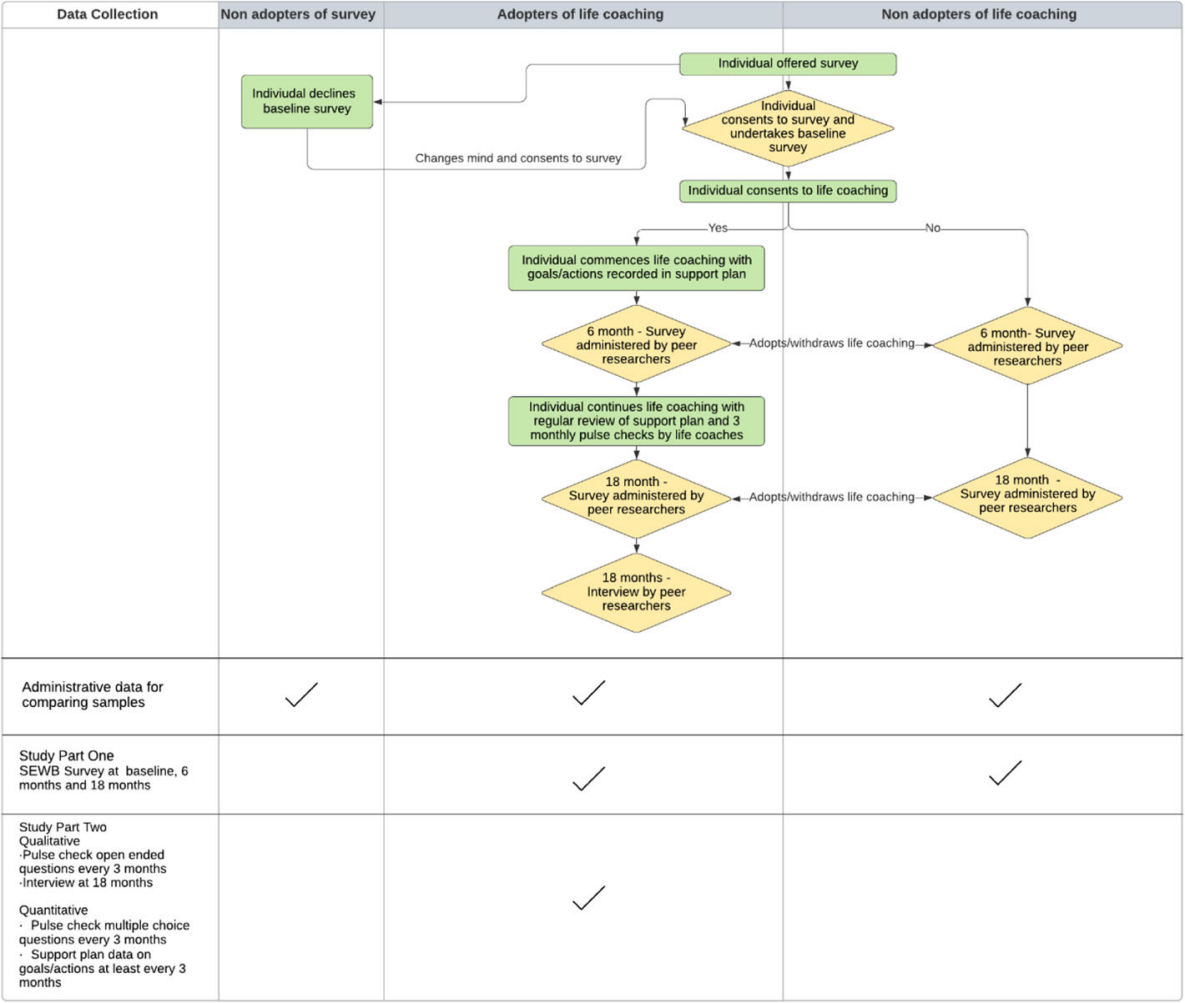
Data collection schema

Peer researchers will administer surveys for part one of the study, the observation of SEWB, and a final qualitative interview for part two to understand the experience of life coaching. Life coaches will regularly capture data about a tenant’s progress toward their goals for part two. The data collection methods are explained in more detail in the following sections and summarised in Fig. 4.

#### Survey

The MTAL survey, including items examining SEWB, was initially developed and used as part of an Indigenous led First 1000 days Australia initiative to describe the aspirations and needs of Aboriginal and Torres Strait Islander families [31]. The survey was modified to meet the needs of the MTAL project and piloted by an Aboriginal controlled health and community service in the eastern region of Melbourne with 22 local community members. The survey was then tailored to the AHV context by AHV staff and researchers to reflect the shift in focus from parenting to people who are tenants of AHV. The revised survey was piloted with five Aboriginal and Torres Strait Islander AHV staff. In addition to demographic information (age, Aboriginal and Torres Strait Islander identification, relationship status, postcode) the survey consists of 12 domains as outlined in Table 1.

**Table 1:** Data collection

Data Type	Domain	Key measures
Survey	Education, Employment & Finances	Highest level of education completed, employment status, barriers to employment, spending, bill payments, bill stress
	Family	Number of children birthed/adopted/cared for, dependents/age/relationship**,** Strengths-based parenting tool [32]
	Out of Home Care	Removals/child protection services, age of housing independence, cultural placement plan
	Culture and Community	Connection to culture and community, community attitudes
	Cultural safety	Unfair treatment, unsafe environments
	Service Use	Service use and availability
	Self determination	Choice and control in decision making on issues
	Health	Health Conditions, Euroqual 5D -5 L [33], disability, smoking
	Wellbeing	Kessler-5 [34], Mental Health Continuum [35], Aboriginal Resilience and Recovery Questionnaire [9]
	Housing	Number and type of occupants, household amenities, financial stress due to tenancy
	Family violence	Experience of controlling, unsafe or threatening behaviour [36]
	Goal setting questions	Key categories of goals/aspirations
Pulse Check	Strengths	Key strengths used
	Resources	Use of community resources
	Resilience	Self-efficacy questions from Aboriginal Resilience and Recovery Questionnaire [9] and help seeking question
	Life coaching	Description of experience and impact of life coaching
Support Plan	Goals and actions	Number and type of goal/s, number of action/s, goals and actions achieved/partially achieved/not achieved
Interview	Life coaching	Experience and impact of life coaching
	Strengths	Awareness and use of strengths
	SEWB	Impact of life coaching on SEWB
Administrative Data	Demographic	Age, Gender
	Household	Household make up (adults-no children, elder, young family with preschoolers, older family with school age children) and household type (unit/house)
	Rent	Rent in arrears (Yes/No)
	Complaints	Number of complaints against household

Survey items are based on either established instruments, modified instruments or instruments developed by researchers or through consultation with key stakeholders. The Aboriginal Resilience and Recovery Questionnaire has been developed and validated for use in the Victorian Aboriginal and Torres Strait Islander population [9].

Surveys will be offered to tenants by peer researchers at baseline, 6 and 18 months. At the completion of the survey all participants are given a debriefing statement containing information about services that could offer support if the questions in the survey have caused them any distress. Some participants may be vulnerable to distress due to potential underlying mental health and/or SEWB vulnerabilities, such as experiences of complex or intergenerational trauma [37]. Survey data will be collected using the Qualtrics platform on a tablet and exported for analysis.

#### Support plan and pulse checks

Each life coach will document information about goal category, actions to achieve goals, goal status and goal attainment date in a support plan. Support plan data will be collected on an AHV database and a report extracted and supplied to the researchers for analysis.

A pulse check has been developed by researchers with AHV staff to determine progress with life coaching and will be administered by life coaches every 3 months. The pulse check is a short survey of mainly multiple-choice questions examining strengths and services used to progress towards goals and ratings of self-efficacy and help seeking behaviour. Open ended questions prompt the participant to reflect on their learning and experience of life coaching to date. Pulse check data will be collected via a Qualtrics survey administered by life coaches via a tablet.

#### Interview

At the time of the 18-month survey, peer researchers will invite all life coaching participants to participate in a semi structured interview. The qualitative interview, based on the principles of appreciative inquiry [38], will explore the participants experience of life coaching. Questions are outlined in an interview schedule and will address participant reflections on their experience of life coaching, strengths, skills and resources used, and the impact of life coaching on their life. Data from the semi structured interview will be audio recorded and transcribed.

#### Administrative data

Administrative data will be analysed on all AHV tenants in the three regions to enable comparisons to be made between the AHV tenant population, survey and life coaching participants. AHV routinely collects administrative data for operational purposes as the landlord. Administrative data will include age, gender, household make up, household type. Administrative data on rent in arrears, tenant related maintenance charges and complaints will also be collected as an absence of issues in relation to this data is indicative that tenants are experiencing fewer of the difficulties in life that may have led to these.

#### Data linkage and protection

Data linkage across administration, survey and life coaching data collection tools will occur through an AHV unique tenant identifier. Date of birth and address will also be collected for the survey to enable identity confirmation checks should the peer researcher enter the incorrect unique tenant identifier number.

Data collected for the support plan as part of AHV providing a life coaching service will be stored by AHV as part of their normal client management database. Survey data will be securely stored by the university-based research team along with life coaching pulse check data. Audio recorded qualitative interview data will be identified according to unique identifier and will be collected by peer researchers and submitted to the university researcher for secure storage. Administrative data will be provided to university research team identified only by unique identifier. Data identified by unique identifier, and for the survey also by date of birth and address, will be kept in password protected databases. Access will be restricted to researchers undertaking the analysis.

### Culturally safe and ethical evaluation

The MTAL study aligns with contemporary principles and guidelines of research with Aboriginal and Torres Strait Islander peoples. MTAL follows the lead of Aboriginal and Torres Strait Islander community members and organisations who identified the need to build the evidence base for strength-based interventions. In this way the study respects and acknowledges the rights of Aboriginal and Torres Strait Islander people to design, plan, implement and evaluate their own programs [39, 40]. The survey reflects and captures Aboriginal and Torres Strait Islander knowledge of health and wellbeing [41]. The structure of the study in engaging Aboriginal and Torres Strait Islander peer researchers and life coaches builds the capacity of local people in the three communities to undertake research and evaluation and develop valuable work experience [40]. The processes, intervention models and data generated in the study will be available to be used for the benefit of AHV and other organisations to promote strengths-based initiatives and advocate for funding. The study incorporates periodic opportunities to share evaluation findings and be accountable to local communities of interest [39].

A number of strategies have been developed to minimise the risk of participants being adversely affected by their involvement in the project. A debriefing statement, as previously described, will be provided to participants after survey administration including information about support services. A distress protocol, which includes processes to temporarily or permanently cease the activity, will be implemented should participants exhibit any distress during the survey, life coaching or interview. Participants will be offered a $40 voucher for their time in completing surveys and a $60 voucher for completing both the 18-month survey and interview.

### Statistical analysis

#### Representativeness of study participants

The data analysis will initially involve comparisons to determine the representativeness of the study participants. The first comparison involves examining whether the participants in the initial survey are representative both of all tenants in each of the three regions and of the full tenant population of AHV. AHV administrative data items will be compared for survey and non-survey participants. This comparison will guide the extent to which study findings could be generalised to the population of AHV tenants or tenants in other settings.

The second comparison examines the comparability between participants in the life coaching program and survey participants who declined life coaching. Comprehensive survey data will be used to explore differences between these two groups that could influence outcomes and potentially confound the estimated difference in outcomes between life coaching participants and those who declined. Potential confounders specified a priori will include education level, life stage, employment status, financial stress, family violence, childhood removal from natural family, out of home care, health and wellbeing, housing stability (e.g. absence of rent in arrears) and connection to community and family.

All comparisons will be made using linear and logistic regression for continuous and binary outcomes respectively. Robust estimation will be used to account for the clustering structure within the study, according to similarity (non-independence) of outcomes between people within a region and similarity of outcomes between people receiving life coaching from the same life coach.

#### Outcome comparisons and SEWB over the period of life coaching

The final comparison compares the SEWB of life coaching participants with non-participants. The assessment of outcomes will be made by analysing a range of outcome variables reflecting the holistic nature of SEWB as defined earlier. Several aspects of the program delivery are hypothesised to bring about changes in the SEWB of life coaching participants. These include the total hours spent in life coaching, the period of life coaching, the number of and degree to which participants achieve goals and their related actions. It is anticipated that the type of goal chosen will influence which outcomes variable may change. For example, a goal to improve financial resources would likely result in a reduction in financial stress and perhaps an improvement in wellbeing but may have less impact on other outcomes such as connection to culture.

The predictor and outcome variables of interest are shown in Table 2.

**Table 2:** Predictor and outcome variables

Predictor variables	Outcome variables
Period of life coaching (months)	Employment status
Exposure/dose of life coaching (hours)	Financial stress
Type of goal (category e.g., financial, employment)	Strengths based parenting
Number of goals (not achieved/partially achieved /achieved)	Connection to culture
Number of actions (not achieved/partially achieved/achieved)	Service use
	Health
	Wellbeing - resilience, psychological distress and mental health

Given the non-randomised design, adjusted analyses will be conducted to delineate whether any observed differential outcomes are due to coaching or could be accounted for by initial differences between the people who chose or declined to participate in life coaching. Comparisons will be adjusted for confounders specified a priori (as above) as well as baseline measure corresponding to the outcome under consideration.

For outcomes measured on a continuum (Euroqual 5D-5L, Kessler-5, Mental Health Continuum, and Aboriginal Resilience and Recovery Questionnaire) multilevel growth models [42] will be used to examine within-person change over time within individuals and communities in each region. The models will be extended to examine the effects of family contexts on the growth curves including individual, family and community level social determinants as measured in the surveys.

Exploratory analyses will be conducted to examine whether the effects of life coaching may be modified by the presence of family violence, family structure (e.g., single parent) or age of children (young vs school aged or teenagers).

### Statistical power

Statistical power will be maximised through the application of growth curve models which more efficiently utilise within person repeated measures than traditional statistical methods. Sample sizes approaching at least 100 are preferred for application of this technique [43] thus the envisioned recruitment will enable these detailed analyses to be conducted.

## Discussion

The MTAL study will contribute to advancement of knowledge, with the results being used to advocate for interventions of this type within the communities of interest and with funders.

A limitation of the study is that the sample of people included in the study is not representative of the general population of Aboriginal and Torres Strait Islander people. The sample does, however, reflect generally the population of tenants of community housing for which this intervention is targeted. The degree to which survey and participants reflect the population of AHV community housing tenants will be determined in the comparative analysis. Previous experience of the life coaching intervention at AHV indicate life coaching participants whose life is more stable are more able to engage in life coaching. The study will assist in identifying which populations the intervention may best be extended to through comparisons of the administrative data collected on all AHV tenants.

The COVID-19 pandemic adds a layer of complexity to rolling out this study. While peer researcher can transition from face to face to remote survey administration if the pandemic requires this, this is less desirable for life coaching. In the initial period of life coaching, face to face meetings assist with the essential task of relationship building between the tenant and the life coach. To address this need, life coaches may need to meet with the tenant initially in public spaces.

Another key implementation challenge is the anticipated turnover in peer researcher staff. Local peer researcher staff are engaged on casual contracts to administer the survey in their region when needed. Peer researchers, who are themselves tenants of AHV, are likely to experience change in their life circumstances during the course of the 18 month roll out of surveys. To manage this risk, more peer researchers will be trained than needed for the survey roll out to allow for loss to follow-up. In addition, peer researchers may be able to undertake surveys in other regions if required.

The result of the study will be discussed periodically throughout the roll out of the intervention through community and stakeholder forums. Dissemination of final results will be subject to the approval of the governance committee. Results will be disseminated to communities via AHV newsletters and meetings, more broadly through peer reviewed journals and conference presentations and via briefs to policy makers and funders where relevant. Publications will acknowledge all participants including AHV staff, peer researchers and life coaches involved in the intervention.

The MTAL study will build the evidence base for strengths-based approaches to improving the social and emotional wellbeing of Aboriginal and Torres Strait Islander people. The study will achieve this through evaluating an initiative developed within an Aboriginal controlled organisation and overseen by a governance committee of Aboriginal and Torres Strait Islander and non-Indigenous researchers experienced in ensuring the study aligns with key principles of empowerment and self-determination of Indigenous communities through the research process.

## Data Availability

Not applicable.

## Abbreviations

AHV: Aboriginal Housing Victoria
COVID − 19: 2019 novel coronavirus
MTAL: More Than a Landlord
SEWB: Social and emotional wellbeing
